# Downregulation of P300/CBP-Associated Factor Attenuates Myocardial Ischemia-Reperfusion Injury Via Inhibiting Autophagy

**DOI:** 10.7150/ijms.44604

**Published:** 2020-05-18

**Authors:** Liqiang Qiu, Changwu Xu, Hao Xia, Jing Chen, Huafen Liu, Hong Jiang

**Affiliations:** 1 Department of Cardiology, Renmin Hospital of Wuhan University, Wuhan 430060, China.; 2 Cardiovascular Research Institute, Wuhan University, Wuhan 430060, China.; 3 Hubei Key Laboratory of Cardiology, Wuhan 430060, China.

**Keywords:** P300/CBP-associated factor, Myocardial ischemia-reperfusion injury, Autophagy, Signaling pathway, Wortmannin

## Abstract

Cardiomyocyte autophagy plays an important role in myocardial ischemia-reperfusion injury (MIRI). P300/CBP-associated factor (PCAF) was involved in the regulation of autophagy. However, the role of PCAF in MIRI is currently unknown. This study was to investigate whether downregulation of PCAF attenuate MIRI. The results showed that the expression of PCAF was significantly increased in MIRI *in vivo* and *in vitro*. Downregulation of PCAF not only inhibited autophagy and damage of H9c2 cells induced by hypoxia-reoxygenation, but also reduced autophagy and myocardial infarct size during myocardial ischemia-reperfusion in rats. In addition, downregulation of PCAF promoted activation of PI3K/Akt/mTOR signaling pathway in cardiomyocytes during hypoxia-reoxygenation. Wortmannin, a PI3K/Akt inhibitor, could abrogate the effects of downregulation of PCAF on cardiomyocytes autophagy. These results demonstrated that downregulation of PCAF alleviated MIRI by inhibiting cardiomyocyte autophagy through PI3K/Akt/mTOR signaling pathway. Thus, PCAF may be a potential target for prevention and treatment of MIRI.

## Introduction

Myocardial infarction has been a leading cause of morbidity and mortality worldwide 1, 2. Percutaneous coronary intervention could effectively restore blood supply to ischemic myocardium, then reduce myocardial infarct size and improve prognosis 3, 4. However, myocardial ischemia-reperfusion injury (MIRI) after revascularization can further aggravate the condition and limit clinical efficacy 5. How to effectively reduce MIRI remains a major challenging research focus.

P300/CBP-associated factor (PCAF), a transcriptional co-activator with intrinsic histone acetyltransferase activity, participates in transcriptional regulation of genes by acetylating histone and non-histone, and is involved in cell differentiation, apoptosis, tumor occurrence and so on 6-8. In addition, studies have shown that PCAF is involved in the transcription of autophagy-related signaling pathway proteins and regulate autophagy 9. It is well known that autophagy is closely related to MIRI 10, 11. In the myocardial ischemia phase, autophagy could protect cardiomyocytes, while during reperfusion phase excessive autophagy is harmful 10, 12, 13. The manipulation of autophagy may be a potential therapeutic strategy to protect against ischemia-reperfusion (I/R)-induced cardiomyocyte death and maintain cardiac function 14. However, whether PCAF could protect MIRI by regulating autophagy is unknown yet. Hence, this study was to investigate whether downregulation of PCAF attenuate MIRI by inhibiting autophagy.

## Materials and methods

### Materials and Agents

The H9c2 cell line was purchased from the Cell Bank of the Chinese Academy of Sciences (China). Wortmannin was purchased from Solarbio (China). Dulbecco's modified Eagle medium F12 (DMEM/F12) was purchased from HyClone (USA). Fetal bovine serum (FBS) was purchased from EVERY GREEN (China). Cell Counting Kit 8 (CCK-8) was obtained from Dojindo Molecular Technologies (Japan). Enzyme-linked immunosorbent assay (ELISA) kits, lactate dehydrogenase (LDH) and creatine kinase (CK) assay commercial kits were obtained from Elabscience Biotechnology Company (China). Primary antibody against glyceraldehyde-phosphate dehydrogenase (GAPDH) was obtained from Beyotime Biotechnology (China). Antibodies against PCAF, total Akt (t-Akt), phosphorylation Akt (Ser473) (P-Akt), total mTOR (t-mTOR), phosphorylation mTOR (P-mTOR), Beclin-1, light chain 3 (LC-3)II were obtained from Cell Signaling Technology (USA).

### Construction of Adenoviral vectors

The specific method for adenovirus construction was reported previously 15. SiRNA sequence against the rat PCAF gene (known as Kat2b, GenBank Accession NC_005108.4) was designed and synthesized by GeneChem (Shanghai, China). A scrambled siRNA was used as a negative control. Sense siRNA sequence for PCAF is 5'-GACAAACTGCCTCTTGAGAAA-3', and 5'-TTCTCCGAACGTGTCACGT-3' for the control. Adenovirus-encoded siRNA against PCAF (Ad-PCAF-RNAi) and control (Ad-GFP) were produced by co-transfecting HEK293 cells according to standard protocols.

### Adenoviral transduction* in vivo* and rat MIRI model

The protocol for animal care was conformed to the Guide for the Care and Use of Laboratory Animals (the National Academies Press, 2011) and the experiment was approved by the Animal Care and Use Committee of Renmin Hospital of Wuhan University. Male SD rats weighing 200-250 g were randomly divided into four groups (n=12 for each group), the sham operation group (Sham group), the myocardial I/R group (I/R group), the myocardial I/R with Ad-GFP group (I/R + Ad-GFP group), and the myocardial I/R with Ad-PCAF RNAi group (I/R + Ad-PCAF RNAi group). Ad-GFP or Ad-PCAF RNAi was transferred or saline was injected into the left ventricular wall of the rat heart as reported previously 16, 17.

Briefly, the SD rats were sedated with isoflurane and anesthetized with pentobarbital (40 mg/kg via intraperitoneal injection), and were intubated orally with 100% oxygen using a rodent ventilator. After left thoracotomy, Ad-GFP (2×10^10^ PFU), Ad-PCAF RNAi (2×10^10^ PFU) or saline in volume of 100 μL was injected into five different sites of the myocardium via a microsyringe. All the five injection sites were situated in the left ventricular anterior wall around the LAD artery. Three days after adenovirus or saline injection, the rat MIRI model was established as previously described 18. Then all rats were re-anesthetized and ventilated with 100% oxygen. A 6-0 silk suture with a curved needle was placed under the origin of the LAD artery and a medical latex tube was located over the LAD artery. Myocardial ischemia was induced by tightening the suture for 30 min. Successful induction of myocardial ischemia was indicated by the presence of ST-segment elevation on electrocardiography. Then loosen the suture to restore coronary circulation. At 24 h post-reperfusion, the rats were re-anesthetized and blood samples were collected through the inferior caval vein. The rats were then sacrificed by air embolism method and myocardial tissue around the infarct border was obtained for further analysis. Rats in the sham group underwent the same procedure but were not subjected to LAD coronary artery ligation.

### Evans blue-triphenyltetrazolium chloride (TTC) staining

Evans blue-TTC double staining was performed immediately after I/R, as previously described 19. Briefly, at the end of 24 h of reperfusion, 2 mL of 1% Evans blue solution was injected through the femoral vein. Hearts were excised, and sliced into 1-mm thick cross sections. The heart sections were then incubated with a 1% triphenyltetrazolium chloride (TTC) solution at 37°C for 15 min. Then the sections were photographed digitally. The non-ischemic portion of the heart was stained dark blue, viable tissue within the area at risk (AAR) was stained bright red, and infarcted area was white or light yellow. The images were analyzed using Image J, and the myocardial infarct size was expressed as a percentage of the infarct area over the total left ventricular area (Inf/LV), and the percentage of the infarct area over AAR area (Inf/AAR).

### Hematoxylin and eosin staining

Heart tissues were obtained at 24 h post-reperfusion and immediately fixed in 4.0% paraformaldehyde for 24 h. After dehydration at room temperature by a graded alcohol series, specimen slices were embedded in paraffin and sectioned at 4 μm. The sections were then stained with hematoxylin-eosin (H&E), photographed, and analyzed under a light microscope (Leica Microsystems, Wetzlar, Germany).

### Transmission electron microscopy (TEM)

Autophagosomes were detected by TEM as previously described 20, 21. In brief, after reperfusion, fractions (1 mm^3^) from fresh left ventricle tissues were prefixed in a solution of 2.5% glutaraldehyde, and then fixed in 1% osmium tetroxide, dehydrated in increasing series of alcohols, and embedded in epoxy resin. For the* in vitro* study, each group of cells was cultured with the related treatment. The cells were washed, digested, centrifugalized, collected, and fixed by 2.5% glutaraldehyde for 24 h at 4 ℃. Next, the cells were embedded in Epon 812 Resin following dehydration in a series of increasing concentrations of acetone. Lastly, the samples were cut into 1 μm sections and the sections were stained with sodium acetate and lead citrate. Ultrathin sections were observed under a transmission electron microscope (JEM-1400Plus; JEOL, Tokyo, Japan).

### Cell culture and adenoviral infection of H9c2 cells

An H9c2 cell line was cultured in DMEM/F12 complemented with 10% FBS, 1% penicillin and streptomycin at 37 °C under air with 5% carbon dioxide. For PCAF downregulation assay, sub-confluent H9c2 cells were incubated with Ad-PCAF-RNAi or Ad-GFP at the multiplicities of infection (MOI) of 30 in serum-free media (SFM). After 4 h of incubation, the medium was removed, and cells were cultured in DMEM/F12 containing 10% FBS.

### Establishment of the hypoxia-reoxygenation (H/R) model

To establish the hypoxia condition of the H/R model, the H9c2 cells were exposed to hypoxia for 6h using glucose-free and FBS-free DMEM/F12 buffer in a hypoxia incubator suffused with 5% CO_2_, 1% O_2_, and 94% N_2_ at 37 ℃. Then, the medium was changed to 10% FBS DMEM/F12 under normal conditions to reoxygenation for 24 h.

### CCK-8 assay

Cell viability was assessed with the CCK-8 assay as described previously 22. Briefly, H9c2 cells were seeded in 96-well plates at a density of 10,000 cells per well in 100 µL culture medium. After adherent culture overnight, Ad-PCAF-RNAi or Ad-GFP was transfected. Then the cells were subjected to H/R. After the model was completed, 10 µL of the CCK-8 reagent was added and the OD value was measured at 450 nm using a microplate spectrophotometer (Infinite 200 Pro; Tecan Group Ltd., Switzerland).

### LDH and CK detection

Blood samples and cell supernatants were collected respectively. LDH and CK assay commercial kits were used to analyze enzyme activity levels. All procedures were performed in accordance with the manufacturer's instructions. The levels of CK and LDH were presented as fold changes relative to the sham or control sample.

### Western blot

Total cellular proteins were extracted using a lysis buffer. After quantification using a BCA kit, an equal amount of 30 µg proteins were loaded to a 12% SDS-polyacrylamide gel electrophoresis (SDS-PAGE) and then transferred to polyvinylidene fluoride (PVDF) for 2 h at 200 mA using a transfer system. The membranes were thereafter blocked with 5% skim milk in tris buffered solution containing 1% tween 20 (TBST) for 2 h. After washing with PBST for three times, the membranes were incubated with corresponding primary antibodies overnight at 4℃. After washing with PBST, The membranes were developed with secondary antibodies at room temperature for 1 h. Finally, the membrane was incubated with ECL solution and then exposed using Bio-Rad ChemiDoc Touch Imaging System.

### Quantitative real-time PCR (Q-PCR)

Total RNA was extracted from myocardial tissue using a commercial RNA isolation kit (Qiagen, Germany). 3.6 μg of RNA was reverse transcribed into cDNA using superscript® III first-strand synthesis system (Invitrogen, USA) according to the manufacturer's instructions. Real-time PCR was performed using Power SYBR Green PCR master mix (Bio-Rad, USA). Data were normalized to GAPDH gene expression, and calculated using the comparative quantification method (2^-ΔΔCt^). Primers for amplifying rat genes were as follows:

GAPDH forward primer, 5'-ACAGCAACAGGGTGGTGGAC-3' and reverse primer, 5'-TTTGAGGGTGCAGCGAACTT-3';

PCAF forward primer, 5'-TTTCTGTCAGCACATTCGGC-3' and reverse primer, 5'-GGGTTTTGTGTTTCGGGTCA -3'.

### Statistical analysis

Data were presented as mean ± standard deviation (SD) and the statistical analysis was performed with Statistical Product and Service Solutions 22.0 software. Analysis of variance (ANOVA) was used for multiple comparisons and least significant difference t test (LSD) for post hoc tests. A *p* value less than 0.05 was considered statistically significant.

## Results

### I/R increases the expression of PCAF* in vivo*

**Fig 1:**
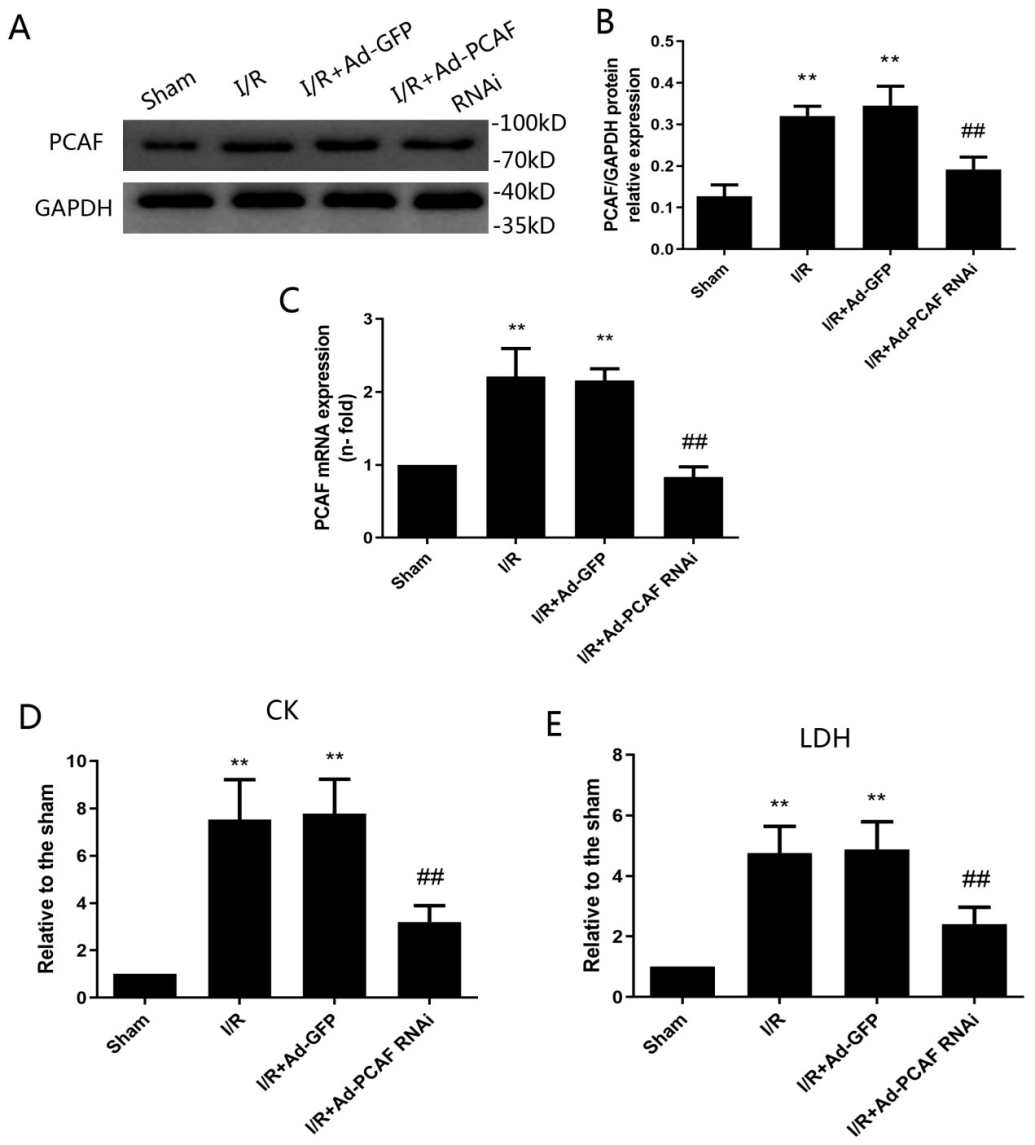
** Ischemia-reperfusion (I/R) increases the expression of PCAF in vivo and downregulation of PCAF inhibits myocardial enzyme levels.** (A-B) Ischemia-reperfusion (I/R) increased the protein expression of PCAF detected by Western blot analysis (n=6). (C) I/R increased the mRNA expression of PCAF detected by Q-PCR (n=6). (D) The activity of serum creatine kinase(CK) (n=5). (E) The activity of serum lactate dehydrogenase(LDH) (n=5).^**^p<0.01 vs. the sham operation group (Sham group); ^##^*p*< 0.01 vs. the myocardial I/R with Ad-GFP group (I/R + Ad-GFP group). I/R + Ad-PCAF RNAi group, the myocardial I/R with Ad-PCAF RNAi group.

To determine whether PCAF involves in the process of MIRI, the protein and mRNA expression of PCAF was explored* in vivo*. As shown in Fig. 1A-B, compared to the sham group, the protein expression of PCAF in the I/R group was increased by 1.5-fold (*p*<0.05). However, the transduction of Ad-PCAF RNAi into the myocardium prior to I/R markedly reduced the PCAF protein expression compared to I/R + Ad-GFP group (0.191±0.023 vs. 0.345±0.035, *p*<0.05, Fig. 1A-B). Similar results were observed at mRNA level (*p*<0.01, Fig. 1C).

### Downregulation of PCAF attenuates MIRI

**Fig 2:**
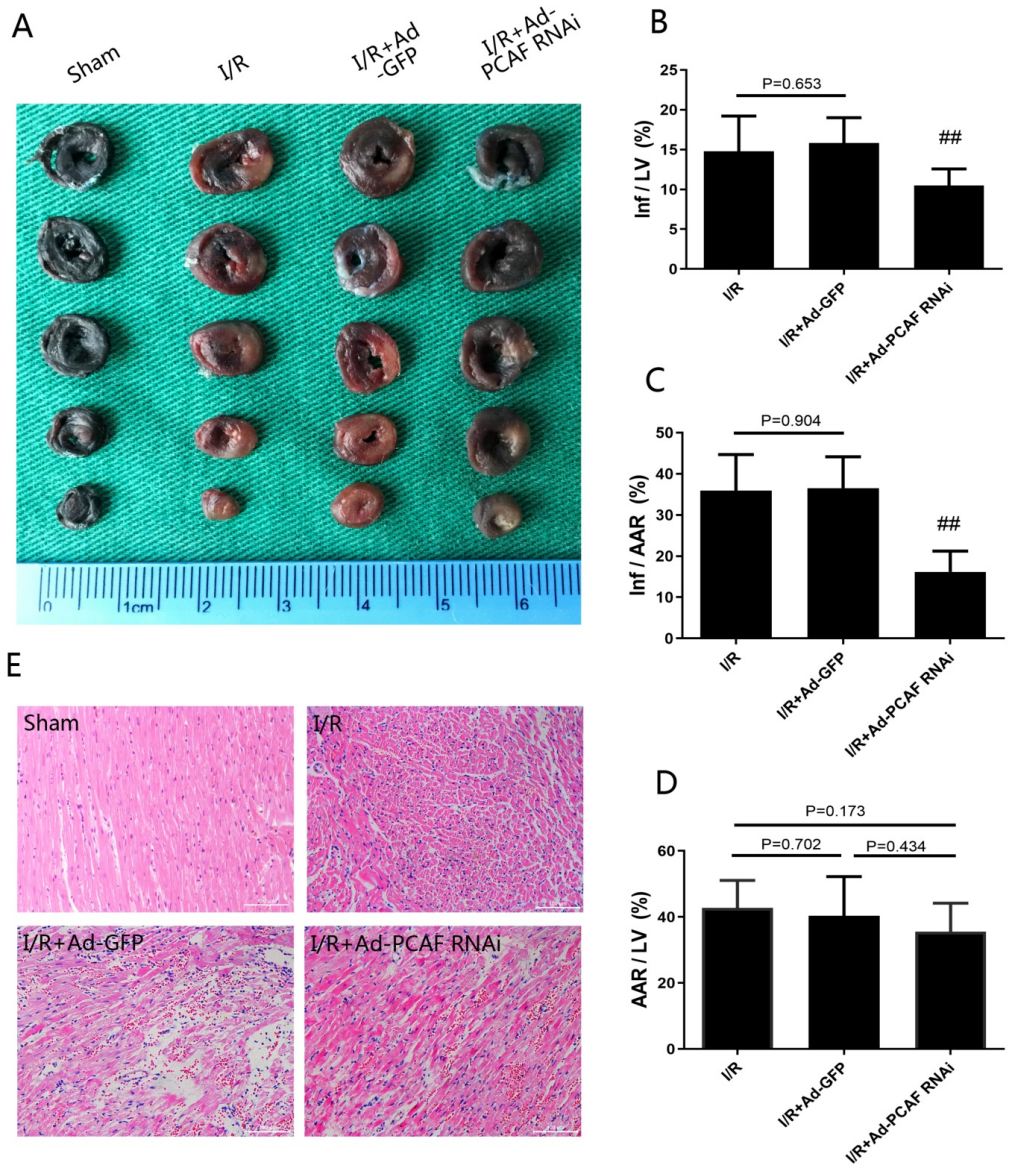
** Downregulation of PCAF attenuates myocardial ischemia-reperfusion injury (MIRI).** (A-D) Evans blue-TTC double staining was used to detect the myocardial infarct size in each group (n=6). (E) Representative images of the rat myocardium under a light microscope with H&E staining (original magnification, x100) (n=6). ^##^*p*< 0.01 vs. I/R + Ad-GFP group.

Infarct size and area at risk for infarction were determined by Evans blue-TTC staining (Fig. 2A). Downregulation of PCAF reduced infarct area normalized to area at total left ventricular (Inf/LV) by 33.7% (*p*<0.01, Fig. 2B). Similar finding was observed when area at infarct was normalized to area at risk (Inf/AAR), which was reduced by around 55.7% (*p*<0.01, Fig. 2C), while the area at risk over total left ventricular (AAR/LV) were not significantly different among groups (*p*>0.05, Fig. 2D). HE staining results showed that the myocardial tissue of the sham group was arranged orderly, without remarkable necrotic cardiomyocytes and inflammatory cells infiltration. In the I/R group and I/R+Ad-GFP group, the myocardial tissue was disordered, as enlarged interstitial space and a large number of inflammatory cells infiltration presented. Compared with the I/R+Ad-GFP group, the myocardial tissue arrangement and the enlarged interstitial space were significantly improved in the I/R+Ad-PCAF RNAi group, and the necrotic cardiomyocytes and the surrounding inflammatory cells infiltration were significantly reduced (Fig. 2E). Meanwhile, compared with the I/R+Ad-GFP group, the CK and LDH levels were remarkably decreased in the I/R+Ad-PCAF RNAi group (both *p*<0.01, Fig. 1D-E).

### Downregulation of PCAF attenuates I/R-induced autophagy dysfunction

**Fig 3:**
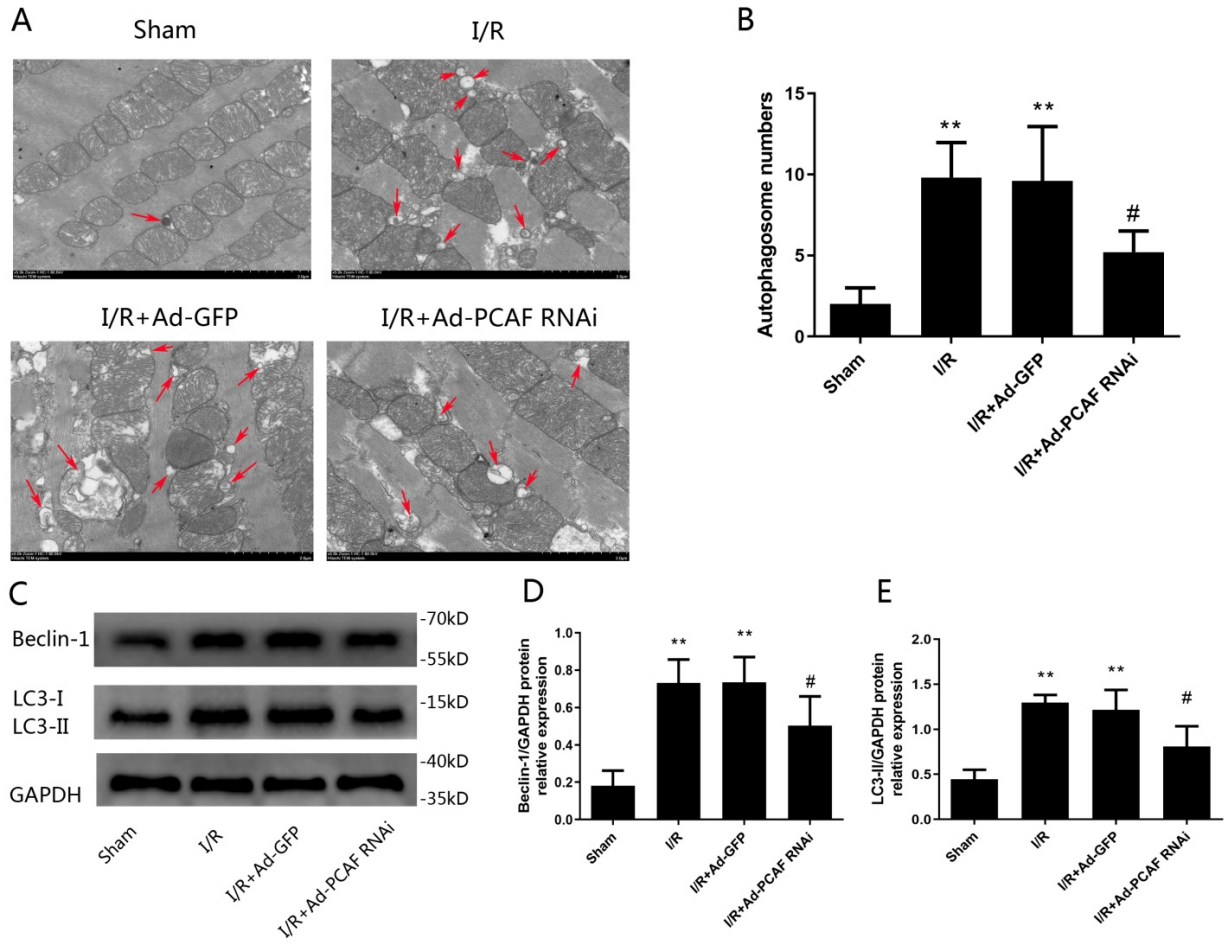
** Downregulation of PCAF attenuates I/R-induced autophagy.** (A-B) The number of autophagosomes in each group was observed under transmission electron microscope (n=5). Scale bar represents 2µm. (C-E) Western blot analysis of Beclin-1 and LC3-II protein levels (n=6). ^**^p<0.01 vs. Sham group; *^#^p*<0.05 vs. I/R + Ad-GFP group.

Previous studies have suggested that autophagy dysfunction is involved in nonapoptotic cell death by I/R 23. Therefore, we further investigated the effects of downregulation of PCAF on autophagy in the I/R myocardium. As shown in Fig. 3A, TEM showed that there were a large number of autophagosomes in the rat heart of I/R and IR+Ad-GFP groups, while downregulation of PCAF reduced autophagosome abundance (Fig. 3B). To further confirm that PCAF was involved in the regulation of autophagic activity, we measured levels of two well-known autophagy markers, LC3-II and Beclin-1. Beclin-1 is an autophagy-related protein with an essential role in the reperfusion-mediated autophagy 24-26. As shown in Fig. 3C, the protein levels of Beclin-1 and LC3-II were increased in all three I/R groups of myocardial samples relative to the Sham group. However, downregulation of PCAF significantly reduced the expression of Beclin-1 and LC3-II by 31.5% and 33.3%, respectively (both *p*<0.05, Fig. 3D-E).

### Downregulation of PCAF alleviates injury during H/R in H9c2 cells

**Fig 4:**
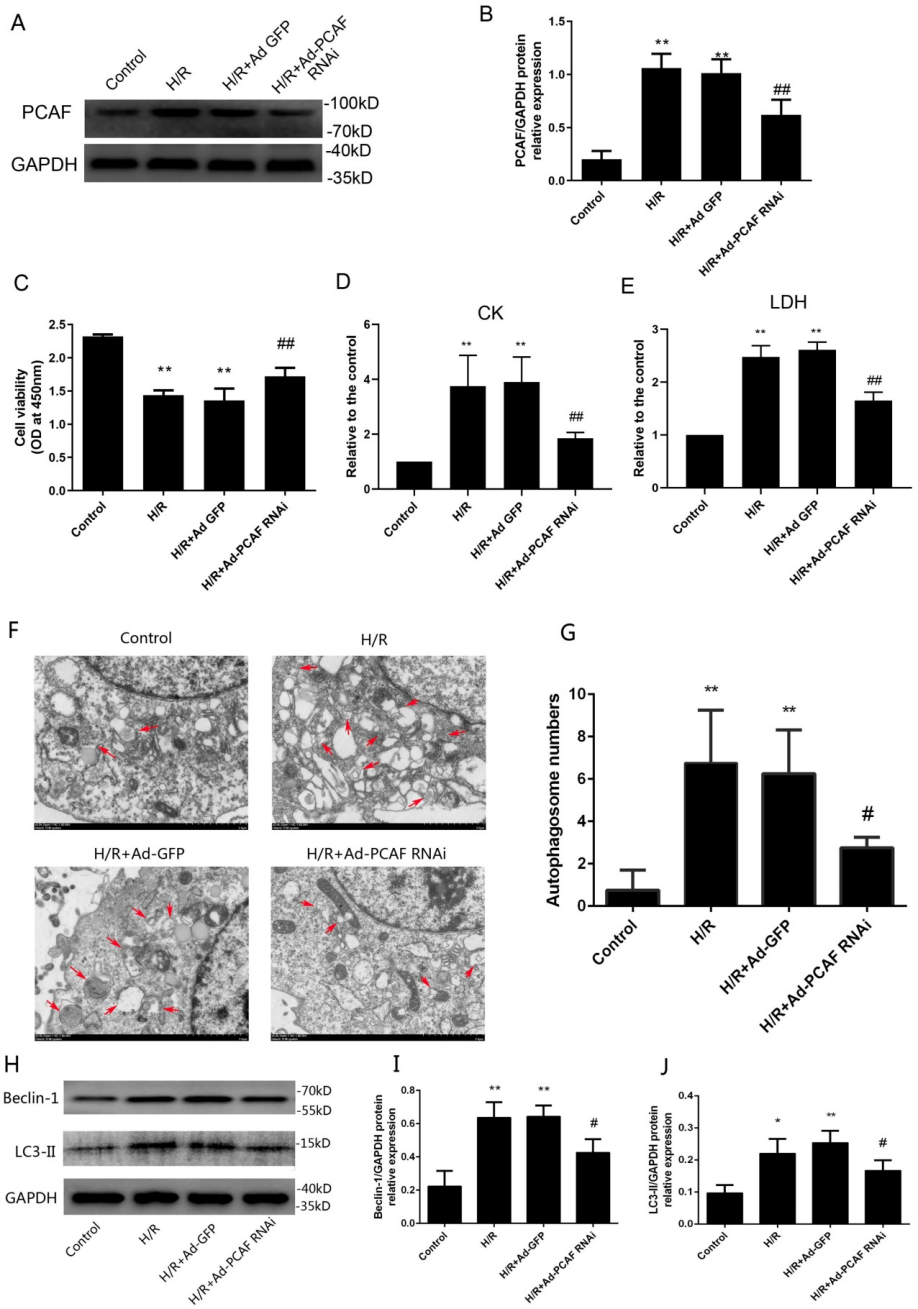
** Downregulation of PCAF reduce the damage of H9c2 cell and attenuates H9c2 cell excessive autophagy during reperfusion.** (A-B) Western blot analysis of PCAF expression and quantitative analysis of the relative protein levels (n=6). (C) CCK-8 assay was used to detect the H9c2 cells viability (n=6). (D-E) ELISA assay to detect the levels of myocardial enzymes (CK, LDH) in the culture supernatant (n=5). (F-G) The number of autophagosomes in each group was observed under transmission electron microscope (n=5). Scale bar represents 2µm. (H-J) Western blot analysis of Beclin-1 and LC3-II protein levels (n=6). ^*^p<0.05,^**^p<0.01 vs. Control group; *^#^p*<0.05,^##^*p*< 0.01 vs. the H9c2 cells H/R with Ad-GFP group (H/R + Ad-GFP group). H/R + Ad-PCAF RNAi group, the H9c2 cells I/R with Ad-PCAF RNAi group.

H/R increased the expression of PCAF and the transduction of Ad-PCAF RNAi into the H9c2 cells markedly reduced the PCAF protein expression compared to H/R +Ad-GFP group (*p*<0.01, Fig. 4A-B). Meanwhile, the cell viability was lower in the H/R and H/R+Ad-GFP groups than in the control group, while downregulation of PCAF markedly increased the cell viability (Fig. 4C). In addition, the levels of CK and LDH in the H/R and H/R+Ad-GFP groups were increased, while downregulation of PCAF decreased the levels of CK and LDH (both *p*<0.01, Fig. 4D-E).

### Downregulation of PCAF attenuates autophagy during H/R in H9c2 cells

The effect of downregulation of PCAF on autophagy activity in H9c2 cells induced by H/R was explored. The number of autophagosomes in H/R group were significantly increased, while downregulation of PCAF remarkably inhibited the increased of autophagosomes (*p*<0.05, Fig. 4F-G). At the same time, we detected the expression of autophagy-related proteins, Beclin-1 and LC3-II. Results showed that Beclin-1 and LC3-II protein levels were increased in H/R group. However, downregulation of PCAF could significantly inhibit the above-mentioned increase (both *p*<0.05, Fig. 4H-J).

### PI3K/Akt/mTOR signaling involves in the regulation of autophagy by downregulation of PCAF

**Fig 5:**
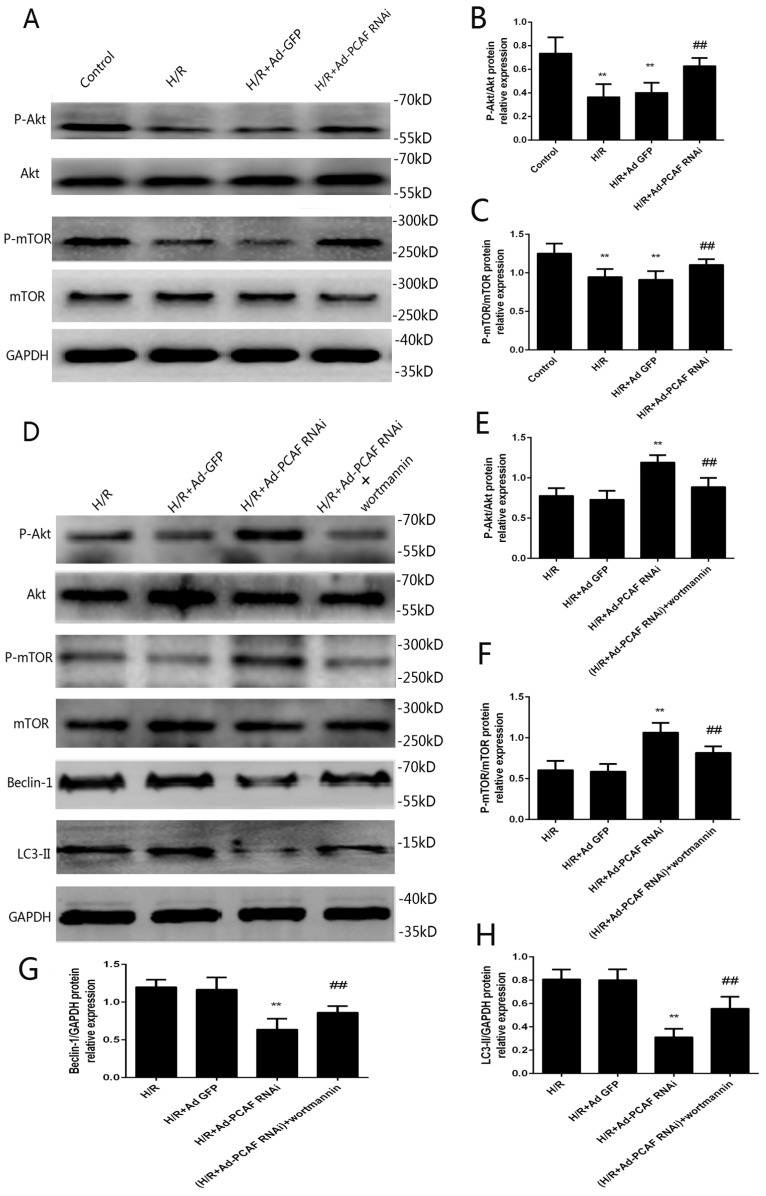
** Involvement of PI3K/Akt/mTOR signaling pathway in the effects of downregulation of PCAF on myocardial autophagy.** (A) Representative gel bolts depicting respective protein expression using specific antibodies. (B-C) Quantitative analysis of the relative protein levels of phosphorylated Akt (p-Akt) and phosphorylated mammalian target of rapamycin (p-mTOR) (n=6). ^**^p<0.01 vs. Control group; ^##^*p*< 0.01 vs. H/R + Ad-GFP group. (D) H9c2 cells were subjected to 6h hypoxia and 24h reoxygenation in vitro with or without the pretreatment of the PI3K/Akt inhibitor Wortmannin (100 nM) for 5 min. Insets: Representative gel bolts depicting respective protein expression using specific antibodies. (E-H) Quantitative analysis of the relative protein levels of p-Akt, p-mTOR, Beclin-1 and LC3-II (n=6). ^**^p<0.01 vs. H/R + Ad-GFP group; ^##^*p*< 0.01 vs. H/R + Ad-PCAF RNAi group.

**Fig 6:**
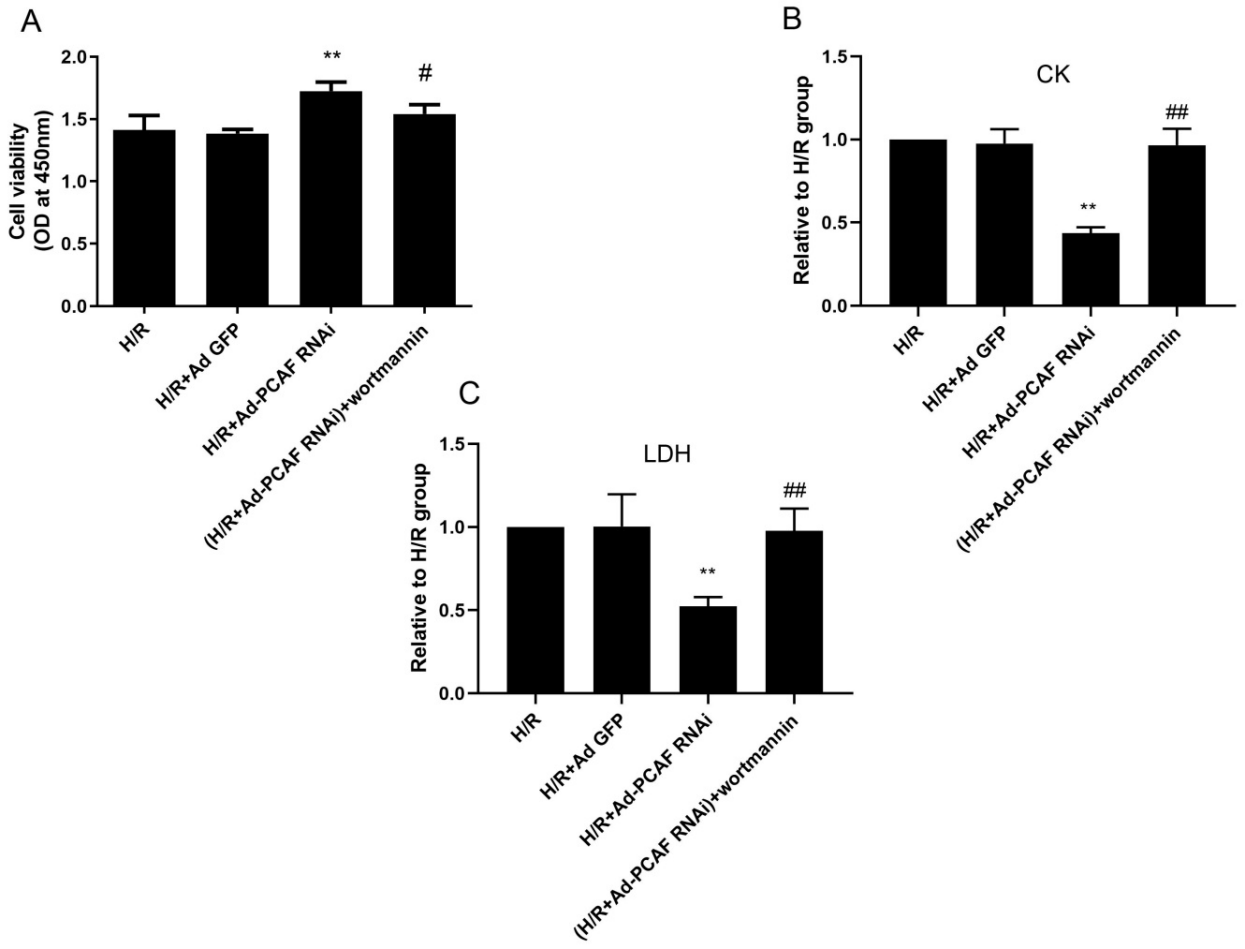
** Involvement of PI3K/Akt/mTOR signaling pathway in the effects of downregulation of PCAF on myocardial damage.** H9c2 cells were subjected to 6h hypoxia and 24h reoxygenation in vitro with or without the pretreatment of the PI3K/Akt inhibitor Wortmannin (100 nM) for 5 min. (A) CCK-8 assay was used to detect the H9c2 cells viability (n=6). (B-C) ELISA assay to detect the levels of myocardial enzymes (CK, LDH) in the culture supernatant (n=5). ^**^p<0.01 vs. H/R + Ad-GFP group; ^#^*p*<0.05,^##^*p*< 0.01 vs. H/R + Ad-PCAF RNAi group.

PI3K/Akt/mTOR signaling is a well-recognized pathway to regulate autophagy 1, 27. To investigate the molecular mechanisms of downregulation of PCAF on autophagy during H/R, the activation levels of Akt and mTOR were examined. As shown in Fig. 5A-C, p-Akt/Akt and p-mTOR/mTOR levels were significantly increased in the H/R+Ad-PCAF RNAi group. To further determine the role of the PI3K/Akt/mTOR signaling pathway the regulation of autophagy by downregulation of PCAF, H9c2 cells were pretreated with 100 nM Wortmannin, a PI3K/Akt inhibitor. As shown in Fig. 5D-H, Wortmannin reduced the activation level of mTOR and enhanced autophagic signaling, which was indicated by decreased p-mTOR and increased Beclin-1 and LC3 II levels. In addition, we further tested cell viability and the levels of CK and LDH in the culture supernatant. Compared to those in the H/R+Ad-PCAF RNAi group, the activity of the cells was significantly decreased and the level of CK and LDH were significantly increased after pretreatment with Wortmannin (all *p*<0.05, Fig. 6A-C).

## Discussion

MIRI has become a key factor that affecting the efficacy of reperfusion therapy 28, 29. Therefore, the study of strategies to reduce MIRI has important clinical significance for the treatment of acute myocardial infarction. To our knowledge, this is the first study to demonstrate that downregulation of PCAF could significantly inhibit excessive autophagy, thereby protecting cardiomyocytes from MIRI. Our results suggested that inhibition of excessive autophagy by downregulation of PCAF attributed to activation of the PI3K/Akt/mTOR signaling pathway.

PCAF is an important member of the GCN5-related N-terminal acetyltransferase family, which has histone acetyltransferase activity and plays an important role in regulating various cellular physiological activities including cell growth, differentiation, apoptosis, and so on 30, 31. Jia et al. found that PCAF is downregulated in hepatocellular carcinoma (HCC) tissues, and overexpression of PCAF in HCC cells can promote autophagy and cause tumor cell death 9. The present study showed the expression of PCAF is effectively downregulated by Ad-PCAF siRNA *in vivo* and *in vitro* (Fig. S1), and downregulation of PCAF could remarkably inhibit autophagy and protect cardiomyocytes from MIRI.

Autophagy is a prevalent physiological process in cells, which is indispensable in the growth, differentiation and response to external irritations and plays an important role in the occurrence and development of diseases 32-34. A large number of studies demonstrated that autophagy is involved in MIRI 35, 36. Moderate autophagy could protect cardiomyocytes from injure under the stimulation of ischemia-reperfusion, while excessive autophagy could result in cell damage or death 37-39. In the ischemic phase, myocardial ischemia and hypoxia can promote autophagy and protect the ischemic myocardium. However, excessive autophagy during reperfusion phase often causes myocardial damage, and even cardiomyocyte death 40-42. Inhibition of excessive autophagy activity in cardiomyocytes during reperfusion phase can significantly reduce cardiomyocyte apoptosis 43, 44. Therefore, it is recognized to inhibit excessive autophagy during reperfusion phase to protect myocardial cells from MIRI. In this study, we found that I/R and H/R induced excessive autophagy in cardiomyocytes and caused significant damage of cardiomyocytes. Meanwhile, downregulation of PCAF inhibited excessive autophagy in myocardial cells during reperfusion and significantly reduced infarct size and myocardial damage.

As a common physiological process, autophagy is often regulated by multiple signaling pathways, of which Akt/mTOR signaling is the most classical 45, 46. Inhibition of Akt/mTOR signaling is recognized as an effective pathway to promote autophagy and promoting Akt/mTOR signaling could inhibit autophagy 27, 47. We found that downregulation of PCAF significantly promoted phosphorylation of Akt and mTOR. At the same time, autophagy activity was significantly inhibited. Using Wortmannin could partly abrogate the effect of downregulation of PCAF on autophagy. These results implied that downregulation of PCAF inhibited autophagy, at least in part, by promoting the PI3K/Akt/mTOR pathway (Fig. S2).

Several limitations need to be noted in this study. First, we used the H9c2 cell line instead of primary cardiomyocytes. Although H9c2 cell line has been widely employed in the study of myocardial injury *in vitro* 48, caution must be taken in interpreting the results due to the obvious difference in cell identity 49. Second, downregulation of PCAF was achieved with adenoviral transduction* in vivo* and* in vitro*. It would be desirable to use a cardiac-specific *pcaf*^-/-^ model. However, such model is not available at this point. Third, further work is needed to explore the mechanism of PCAF on myocardial autophagy and MIRI beyond the PI3K/Akt/mTOR signaling pathway.

In summary, our findings indicated that downregulation of PCAF could attenuate MIRI by inhibiting excessive autophagy through regulating PI3K/Akt/mTOR signaling pathway and inhibition of PCAF may be a potential therapeutic target to protect against MIRI.

## Supplementary Material

Supplementary figures and tables.
