# Supplementary material for: Downregulation of P300/CBP-Associated Factor Attenuates Myocardial Ischemia-Reperfusion Injury Via Inhibiting Autophagy
Source: Int J Med Sci. 2020 May 18;17(9):1196–206. doi: 10.7150/ijms.44604 (PMC7294925; doi:10.7150/ijms.44604)

Fig.S1 The efficiency of PCAF knockdown by Ad-PCAF RNAi *in vitro* and *in vivo*. (A) Western blot analysis of PCAF protein expression in H9c2 cells after adenoviral infection (n=6). (B) Western blot analysis of PCAF protein expression in rat myocardium after adenovirus injection (n=6). $^{**}p<0.01$ vs. Ad-GFP group.

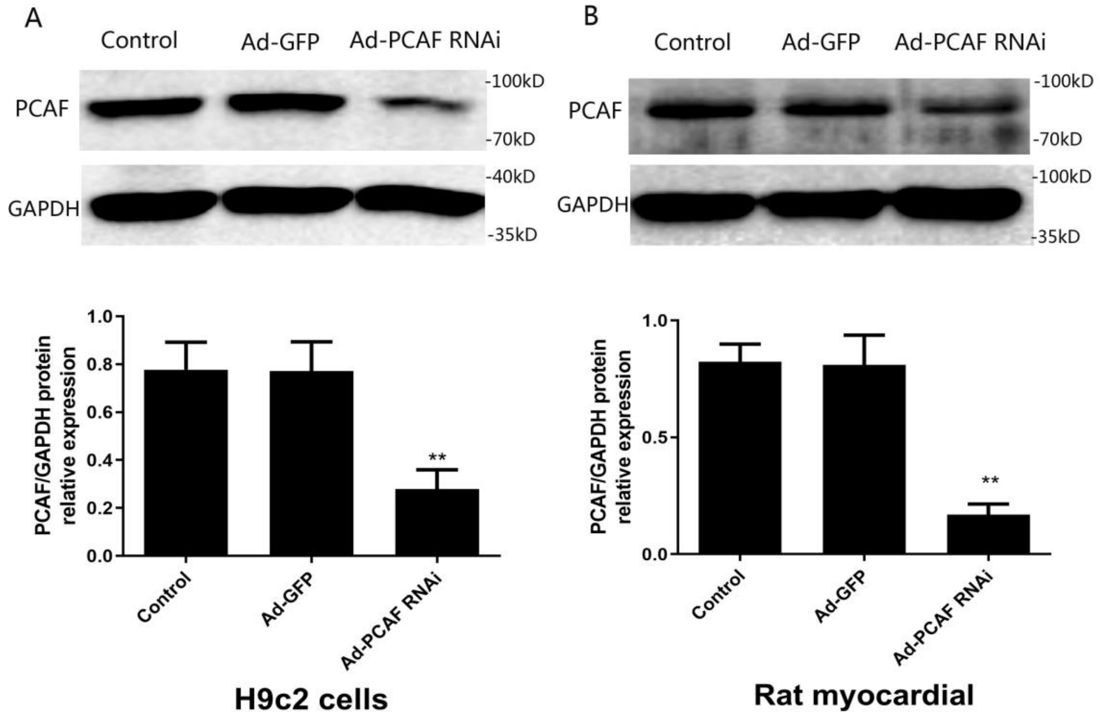

Fig.S2 Graphical abstract for the mechanism of PCAF on myocardial autophagy and myocardial ischemia-reperfusion injury through the PI3K/Akt/mTOR signaling pathway. I/R, ischemia-reperfusion; H/R, hypoxia-reoxygenation.

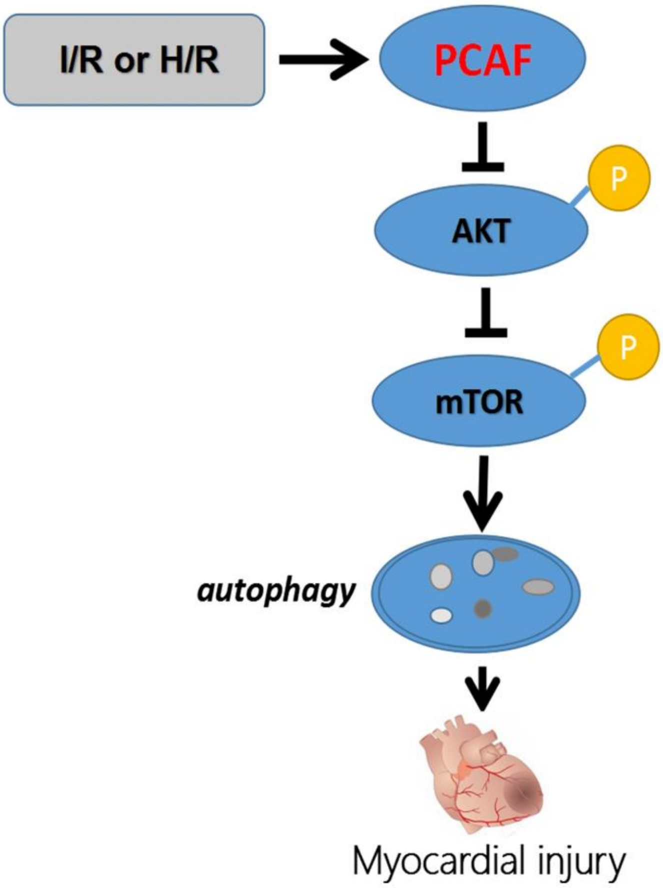

Supplement: Supplementary file 1 — Supplementary figures and tables. [file ijmsv17p1196s1.pdf]
